# Anti-Ro60 Seropositivity Determines Anti-Ro52 Epitope Mapping in Patients With Systemic Sclerosis

**DOI:** 10.3389/fimmu.2018.02835

**Published:** 2018-12-07

**Authors:** Athanasios Gkoutzourelas, Christos Liaskos, Maria G. Mytilinaiou, Theodora Simopoulou, Christina Katsiari, Alexandra Tsirogianni, Dimitrios Daoussis, Thomas Scheper, Wolfgang Meyer, Dimitrios P. Bogdanos, Lazaros I. Sakkas

**Affiliations:** ^1^Department of Rheumatology and Clinical Immunology, Faculty of Medicine, School of Health Sciences, University of Thessaly, Larissa, Greece; ^2^Department of Immunology-Histocompatibility, Evangelismos General Hospital, Athens, Greece; ^3^Department of Rheumatology, Patras University Hospital, Faculty of Medicine, University of Patras Medical School, Patras, Greece; ^4^Institute of Immunology Affiliated to Euroimmun AG, Lübeck, Germany

**Keywords:** autoantibody, autoimmunity, autoimmune rheumatic diseases, epitope, SS-A

## Abstract

Epitope mapping of anti-Ro52 antibodies (Abs) has been extensively studied in patients with Sjögren's syndrome (SjS) and systemic lupus erythematosus (SLE). Comprehensive epitope mapping in systemic sclerosis (SSc), where anti-Ro52 antibodies are also frequently detected, has not been performed. The aim of the present study was to fully characterize Ro52 epitopes in anti-Ro52-positive SSc using Ro52 fragments spanning the full antigen. Further analysis was made according to anti-Ro60 status. Epitope mapping was performed in 43 anti-Ro52-positive SSc patients. Seventy eight anti-Ro52-positive pathological controls, including 20 patients with SjS, 28 patients with SLE, 15 patients with dermatomyositis (DM), and 15 patients with primary biliary cholangitis (PBC), and 20 anti-Ro52-negative healthy individuals as normal controls were also tested. Five recombinant Ro52 fragments [Ro52-1 (aa 1-127), Ro52-2 (aa 125-268), Ro52-3 (aa 268-475), Ro52-4 (aa 57-180), and Ro52-5 (aa 181-320) were used to test reactivity by line-immunoassay and *in house* ELISA. Anti-Ro60 reactivity was tested by ELISA. All anti-Ro52 positive sera reacted with Ro52-2; none recognized Ro52-3. Antibodies against Ro52-1 were less frequently found in SSc than in SjS/SLE (11.6 vs. 41.7%, *p* = 0.001); and antibodies against Ro52-4 were less frequently found in SSc than in SjS/SLE (27.9 vs. 50%, *p* = 0.03). In SSc patients, reactivity against Ro52-1 was more frequent in anti-Ro52+/anti-Ro60+ than in anti-Ro52+/anti-Ro60-patients (33.3 vs. 0%, *p* = 0.003). In this comprehensive analysis of Ro52 epitope mapping in SSc, the coiled coil domain remains the predominant epitope on Ro52. Contrary to SjS and SLE, patients with SSc fail to identify epitopic regions within the N-terminus of the protein, especially if they lack con-current anti-Ro60 reactivity.

## Introduction

Anti-Ro52 antibodies (Abs), along with anti-Ro60 or in isolation, are frequently found in patients with autoimmune rheumatic diseases (AIRDs) (1–4). These autoantibodies (autoAbs), originally described in patients with Sjögren's syndrome (SjS), systemic lupus erythematosus (SLE), are detected in other ARDs, as well as in other organ and non-organ specific autoimmune diseases (1–3, 5–7). For instance, we and others reported the presence of anti-Ro52 Abs in ~20–30% of patients with systemic sclerosis (SSc), making it the third most common antibody (Ab) in this disease (8–10). Ro52, originally considered as potential part of the ribonucleoprotein complex, is now well established as member of the tripartite TRIM family (TRIM21). It has been shown that Ro52 (TRIM21) is a cytosolic Fc receptor, bound with high affinity preferentially to IgG, but also to IgA and IgM intra-cytoplasmic receptor of IgG (11, 12). This ability of Ro52 (TRIM21), for simplicity there after mentioned as Ro52, along with its pleiotropic immunomodulatory properties have led us to appreciate the important role of this antigen in regulation of immune-mediated inflammation and regulation of autoreactive immunity (11, 12).

The exact epitopic regions on Ro52 targeted by antigen-specific autoAbs have been extensively studied in SjS and SLE (13–19), but their characterisation in patients with SSc is ill-defined. In SjS and SLE, anti-Ro52 autoAbs mainly target large polypeptidyl sequences in the coiled coil region of the protein (13–19). Linear short sequences within the corresponding epitopes are subdominantly recognized (20). A recent study by Infantino et al (21), using a set of 5 epitopic regions overlapping the whole sequence, has demonstrated reactivity mainly to aa 125-268. Having access to these Ro52 constructs, we considered that it is worth investigating the B-cell epitopes of Ro52 in patients with SSc. Our findings neither refute nor agree with those obtained in SjS and SLE. When anti-Ro52 Ab-positive SSc patients were divided according to con-current anti-Ro60 Abs, different patterns of epitope recognition were found.

## Material and Methods

### Material

A total of 121 anti Ro52 Ab-positive patients with various autoimmune diseases were analyzed, including 43 patients with SSc (41 females; mean age ±SD: 57.83 ± 12.58 years; disease duration 9.72 ± 7.4 years; 41 ANA positive, median titre 1/160, range 1/80-1/5,120) as the study group (Table 1), 20 patients with SjS (18 females; mean age ±SD: 52 ± 11.2 years), 28 patients with SLE (all females; mean age ±SD: 45 ±14.3 years), 15 with dermatomyositis (DM) (9 females; mean age ±SD: 62.13 ±11.3 years); and 15 patients with primary biliary cholangitis (PBC) (22) (13 females; mean age ±SD: 47.2 ± 13.4 years), as pathological controls. All patients were regularly followed up at the Out-patient Clinic, Department of Rheumatology and Clinical Immunology, University General Hospital of Larissa, in Larissa, Greece (9, 23, 24). A cohort of 10 additional anti-Ro52 Ab-positive SSc patients were also included; these patients were followed up at two other Greek University Hospitals, University of Athens and University of Patras. Diagnosis of SSc was based on the 2013 ACR/EULAR Criteria for the Classification of SSc (25); diagnosis of SjS on the 2016 ACR/EULAR Classification Criteria for primary SjS(26), diagnosis of SLE was based on the 2012 SLICC Criteria (27), and diagnosis of DM was based on the Bohan and Peter Criteria for Polymyositis and Dermatomyositis (28, 29). Diagnosis of PBC was based on the internationally accepted criteria for PBC (22, 30).

**Table 1:** Clinical and immunological characteristics of SSc patients.

	**SScpatients *n* = 35**
**SSctype**	
lcSSc (n,%)	23 (65.7)
dcSSc (n,%)	12 (34.3)
Rodnan skin score (mean ± SD)	7.24 ± 8.3
Pulmonary fibrosis (n,%)	12 (34.3)
Pulmonary arterial hypertension (n,%)	2 (5.7)
Ulcers (n,%)	12 (34.7)
**GI INVOLVEMENT**	
Upper (n,%)	20 (57.1)
Lower (n,%)	0
Both (n,%)	1 (2.9)
Arthritis (n,%)	13 (37.1)
Serositis (n,%)	4 (11.4)
Telangiectasia (n,%)	18 (51.4)
Calcinosis (n,%)	2 (5.7)
Renal crisis (n,%)	0
Overlap syndrome/MCTD (n,%)	8 (25.0)
Dry mouth (n,%)	13 (37.5)
Dry eyes (n,%)	8 (22.9)
Rash (n,%)	7 (20.0)
Acro-osteolysis (n,%)	5 (14.3)
**AUTOANTIBODIES**	
-Scl-70 (n,%)	5 (14.3)
-CENPA (n,%)	14 (40.0)
-CENPB (n,%)	14 (40.0)
-RP11 (n,%)	1 (3.1)
-RP155 (n,%)	3 (9.4)
-Fibrillarin (n,%)	1 (3.1)
-NOR90 (n,%)	3 (9.4)
-Th/To (n,%)	0 (0)
-PM-Scl 100 (n,%)	0 (0)
-PM-Scl 75 (n,%)	2 (6.3)
-Ku (n,%)	4 (12.5)
-PDGFR (n,%)	0 (0)
-Ro52 (n,%)	35 (100)
-Ro60 (n,%)	13 (37.1)
-La (n,%)	5 (14.3)

Fifty anti-Ro52 Ab-negative patients with various AIRDs and other autoimmune diseases, including 12 patients with SSc,10 with SjS, 12 with SLE, 5 with DM, and 11 with PBC, were tested as anti-Ro52 Ab-negative disease controls.

Twenty healthy individuals (all anti-Ro52 Ab-negative) were also tested as normal controls (NCs) (18 females; mean age ±SD: 52.8 ± 10.9 years).

The presence of anti-Ro52 Abs was initially assessed by a line immunoassay (Euroimmun, Lübeck, Germany) and confirmed by an anti-Ro52 specific ELISA (Inova Diagnostics, San Diego, CA).

A written informed consent was obtained by all patients and controls. The study was performed in accordance with the declaration of Helsinki. Patients and NCs participated in the study after approval of the research protocol by the Ethical Committee of the University General Hospital of Larissa, Faculty of Medicine, University of Thessaly, Greece.

### Methods

**Figure 1:**
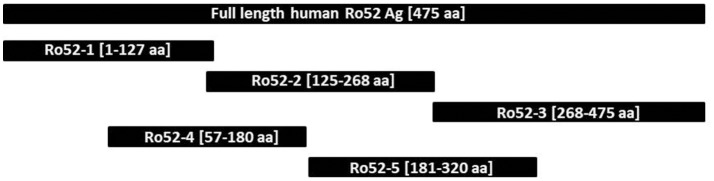
Schematic representation of full length Ro52 antigen and the 5 separate Ro-52 fragments.

Epitope mapping was performed using a specifically-designed line immunoassay containing five recombinant Ro52 fragments expressed in *E coli* [Ro52-1 (aa 1-127), Ro52-2 (aa 125-268), Ro52-3 (aa 268-475), Ro52-4 (aa 57-180), and Ro52-5 (aa 181-320) (Figure 1), as described before (21). Ro-52 full-antigen, expressed with the baculovirus system in insect cells, was used as positive control. Titration experiments were performed to establish optimal conditions of experiments. The final concentration of each fragment was established based on ROC curves using four different concentrations (1, 5, 25, 100 μg/ml) tested in 20 anti-Ro52-positive SSc and 20 anti-Ro52-negative NCs. The final concentration of each fragment that gave specificity up to 94% was as follows: 100 μg/ml for Ro52-1 and 25 μg/ml for all other fragments. The specifically designed line strips were incubated with sera (1:100 dilution) on a rocking platform at room temperature for 30 min (21). After the aspiration of the liquid the strips were washed three times in 1.5 ml wash buffer (Euroimmun) for 5 min. Then strips were incubated in alkaline phosphate-labeled anti-human IgG conjugate (Euroimmun) for 30 min, followed by three 5 min washes (21). Finally, strips were incubated in 5-bromo-4-chloro-3-indolyl-phosphate/nitro blue tetrazolium substrate solution (Euroimmun) for 10 min and then washed with distilled water. After being dried, strips were evaluated by the use of EUROLineScan software (Euroimmun) and results were expressed in arbitrary units (AU/ml), as previously described in detail (9, 21, 31). To determine the cut off values of the *in house* line immunoassays, we tested 70 anti-Ro52 Ab-negative serum samples (39 with various AIRDs, 11 with PBC and 20 NCs, see above). For each fragment the chosen cut off value corresponded to mean+2SD. Based on that, the cut off value was 8 AU/ml for Ro52-1, 10 AU/ml for Ro52-2, 4 AU/ml for Ro52-3, 5 AU/ml for Ro52-4, and 8 AU/ml for Ro52-5 (Supplementary Table 1).

The validity of Ab reactivity to fragments by a line immunoassay was also assessed by an *in house* ELISA using the same Ro52 fragments testing 32 serum samples (12 randomly selected anti-Ro52 Ab positive SSc patients and 20 anti-Ro52 negative SSc patients), as previously described with slight modifications (32–34). Titration experiments were executed to establish optimal conditions of experiments. Briefly, initially each well was incubated at 20°C for 1 h with 200 μl of blocking buffer (2% BSA in PBS), to block non-specific binding). All reagents were purchased by Sigma Aldrich, until otherwise stated. After a washing step (5 times with PBS-0.1% Tween-20), 100 ml of each Ro52 fragment (final concentration: 25 μg/ml) was added to the wells (diluted in PBS containing 0.1% BSA and 0.1% sodium azide) and incubated at 20°C on shaker for 1 h. Washing was repeated (5x) and following that, 100 μl of each samples at 1/200 dilution (in 2% BSA/PBS containing 0.1% sodium azide) was added and incubated at 20°C on a shaker for 1 h. To ensure consistency, two sera were used as reference controls, including a high titre anti-Ro52 Ab serum from an SLE patient, know to strongly react with the Ro52 full protein, and fragments Ro52-1, Ro52-2, Ro52-4, and Ro52-5, and a NC serum used as negative control totally unreactive against the full Ro52 protein and its fragments. The washing step was repeated and 100 μl of conjugate-1/1000 peroxidase-conjugated goat anti-human (IgG) diluted in 2% BSA/PBS were added to each well and incubated at 20°C for 1 h. After washing steps (5x), 100 μl of TMB substrate (3,3', 5,5;-tetramethylbenzidine) was added and incubated in the dark for 10 min. The reaction was terminated by adding 50 μl of H_2_SO_4._ Light absorbance (optical density, OD) was measured against blank well at 450 nm (620 nm as reference wavelength). To determine the cut off value, 20 anti-Ro52 Ab-negative patients (10 with SSc and 10 randomly selected, with other ARDs), were tested with individual Ro52 fragments. Reaction for a given construct exceeded was considered positive when the OD reading of the test serum against the construct exceeded the mean+2SD of the absorbance values of the 32 anti-Ro52 Ab negative controls. To utilize a uniform representation of the absorbance values, the absorbance corresponding to the cut off value was defined as 1 RU/ml.

### Statistical Analysis

All results are expressed as percentages (%). To determine cut off values for the line immunoassay ROC analyses were performed and for each Ro52 fragment ab concentration was chosen for a specificity up to 94%. Mean plus 2SD of values of negative patients were used as cut off for each assay (line immunoblotting, ELISA). Differences between groups were tested by chi-square, two-tailed *t*-test and nonparametric Mann-Whitney test. *p*-values smaller than or equal to 0.05 were considered significant. The statistical calculations were performed with SPSS statistics 22.

## Results

**Figure 2:**
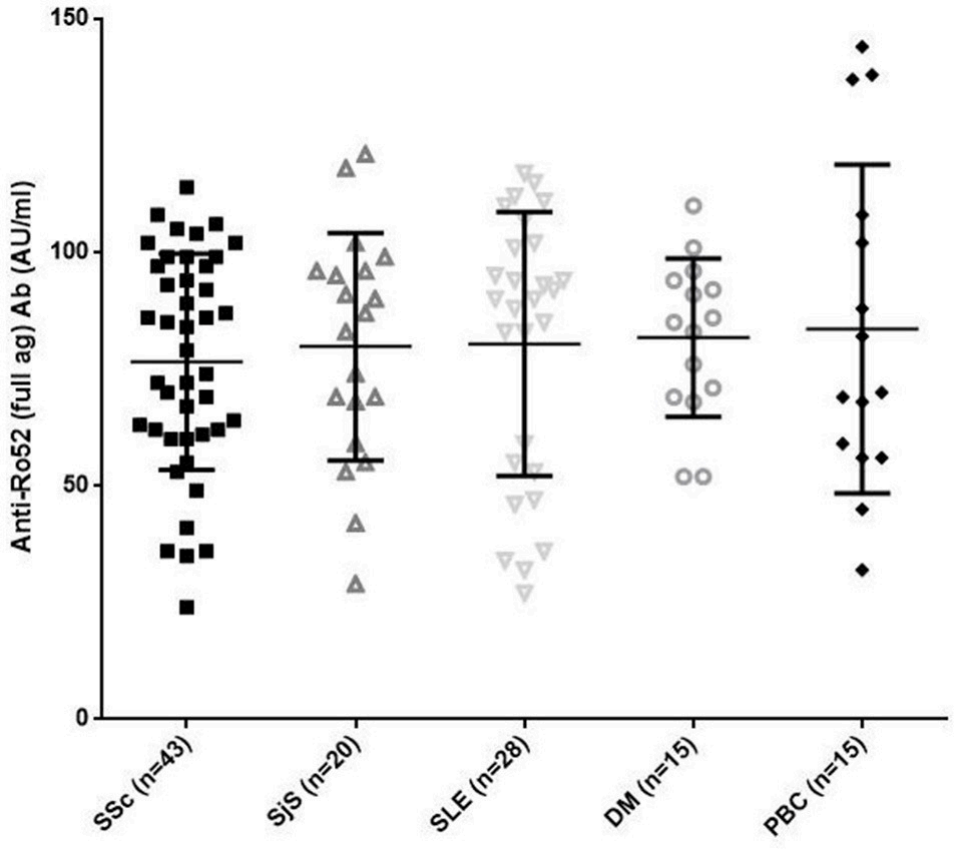
Reactivity in AU/ml against the full length Ro52 antigen in 43 patients with systemic sclerosis (SSc), 20 patients with Sjögren's syndrome (SjS), 28 patients with systemic lupus erythematosus (SLE), 15 patients with DM, and 15 patients with primary biliary cholangitis (PBC). There were no significant differences amongst different diseases. Values are given as symbols. The solid black line at the approximate center of each vertical line is the median. The arms of each line extend with their ends corresponding to 10 and 90% of the values.

All anti-Ro52 Ab-positive patients reacted against the full Ro52 antigen by a line immunoassay without difference in AU/ml among the various diseases (mean ± SD: 76.55 ± 23.13 AU/ml in SSc compared to 79.8 ± 24.3 AU/ml in SjS; 80.39 ± 28.26 AU/ml in SLE; 81.73 ± 16.93 AU/ml in DM and 83.6 ± 35.2 AU/ml in PBC, *p* > 0.05 for all) (Figure 2).

### Frequency of Ro52 Fragment ab Recognition of SSc and Controls

Results of serum reactivity to Ro52 fragments are summarized in Tables 2, 3. Overall, reactivity against fragments Ro52-1, Ro52-2, Ro52-3, Ro52-4, and Ro52-5 in patients with SSc was 11.6, 100, 0, 27.9, and 41.9%, respectively. The respective results in SjS were 40, 100, 0, 40, and 60%; in SLE were 42.9, 100, 0, 57.1, and 57.1%;in DM were 20, 100, 0, 33.3, and 40% and in PBC 6.7, 100, 0, 6.7, and 40%.

**Table 2:** Reactivity against full antigen and Ro52 fragments in various autoimmune diseases.

	**SSc *n* = 43 (%)**	**SjS/SLE (*n* = 48) (%)**	**DM (*n* = 15) (%)**	**PBC (*n* = 15) (%)**	**p (SSc vs. SjS/SLE)**	**p (SSc vs. DM)**	**p (SSc vs. PBC)**	**p (SjS/SLE vs. DM)**	**p (SjS/SLE vs. PBC)**	**p (DM vs. PBC)**
Full antigen	100	100	100	100	NS	NS	NS	NS	NS	NS
Ro52-1	11.6	41.7	20	6.7	**0.001**	NS	NS	NS	**0.01**	NS
Ro52-2	100	100	100	100	NS	NS	NS	NS	NS	NS
Ro52-3	0	0	0	0	NS	NS	NS	NS	NS	NS
Ro52-4	27.9	50.0	33.3	6.7	**0.03**	NS	NS	NS	**0.002**	NS
Ro52-5	41.9	58.3	40	40.0	NS	NS	NS	NS	NS	NS

*SSc, systemic sclerosis; SjS, Sjögrens syndrome; SLE, systemic lupus erythematosus; PBC, primary biliary cholangitis. Significant p-values are indicated in bold*.

**Table 3:** Summary of Ab reactivity against the full antigen and the fragments.

	**SSc *n* = 43 (%)**	**SjS (*n* = 20) (%)**	**SLE (*n* = 28) (%)**	**DM (*n* = 15) (%)**	**PBC (*n* = 15) (%)**	**p (SSc vs. SjS)**	**p (SSc vs. SLE)**	**p (SSc vs. DM)**	**p (SSc vs. PBC)**	**p (SjS vs. SLE)**	**p (SjS vs. DM)**	**p (SjS vs. PBC)**	**p (SLE vs. PBC)**	**p (DM vs. PBC)**
Full antigen	100	100	100	100	100	NS	NS	NS	NS	NS	NS	NS	NS	NS
Ro52-1	11.6	40.0	42.9	20	6.7	**0.009**	**0.002**	NS	NS	NS	NS	**0.04**	**0.02**	NS
Ro52-2	100	100	100	100	100	NS	NS	NS	NS	NS	NS	NS	NS	NS
Ro52-3	0	0	0	0	0	NS	NS	NS	NS	NS	NS	NS	NS	NS
Ro52-4	27.9	40.0	57.1	33.3	6.7	NS	**0.014**	NS	NS	NS	NS	**0.025**	**0.001**	NS
Ro52-5	41.9	60.0	57.1	40	40.0	NS	NS	NS	NS	NS	NS	NS	NS	NS

*SSc, systemic sclerosis; SjS, Sjögren's syndrome; SLE, systemic lupus erythematosus; PBC, primary biliary cholangitis. Significant p-values are indicated in bold*.

According to individual disease, Ab reactivity against fragments Ro52-1, Ro52-4, and Ro52-5 were as follows (SjS and SLE are grouped together as their reactivities to different Ro52 fragments were similar): Abs against Ro52-1 were less frequent in SSc than in SjS/SLE (5/43 [11.6%] vs. 20/48 [41.7%], *p* = 0.001); Abs against Ro52-4 were less frequent in SSc than in SjS/SLE (12/43 [27.9%] vs. 24/48 [50%], *p* = 0.03). In addition, Abs against Ro52-1 were also more frequent in SjS/SLE than in PBC (20/48 [41.7%] vs. 1/15 [6.7%], *p* = 0.01) and Abs against Ro52-4 were more frequent in SjS/SLE than in PBC (24/48 [50%] vs. 1/15 [6.7%], *p* = 0.002) (Table 2).

Comparison of reactivities against Ro52 fragments between SSc and separate SLE or SjS patient groups are shown in Table 3. In particular, SSc patients were less frequently reactive against Ro52-1 than SjS (5/43 [11.6%] vs. 8/20 [40.0%], *p* = 0.0095) and SLE patients (5/43 [20.9%] vs. 12/28 [42.9%], *p* = 0.002). PBC patients were also less frequently reactive against Ro52-1 than SjS (1/15 [6.7%] vs. 8/20 [40.0%], *p* = 0.04) and SLE patients (1/15 [6.7%] vs. 16/28 [57.1%], *p* = 0.02). Moreover, SSc patients were less frequently reactive against Ro52-4 than SLE (12/43 [27.9%] vs. 16/28 [57.1%], *p* = 0.014) and this was also the case for PBC compared to SjS (1/15 [6.7%] vs. 8/20 [40.0%], *p* = 0.025) and SLE patients (1/15 [6.7%] vs. 16/28 [571%], *p* = 0.001) (Table 3).

Ab reactivity to individual Ro52 fragments by line immunoassay correlated with Ab binding of the same fragments when tested by *in house* ELISA (*r* = 0.95, *p* < 0.001 for Ro52-1; *r* = 0.783, *p* = 0.003 for Ro52-2; *r* = 0.485, *p* = 0.11 for Ro52-3; *r* = 0.729, *p* = 0.007 for Ro52-4; *r* = 0.784, *p* = 0.003 for Ro52-5) (Supplementary Figure 1). All sera tested negative for Ro52 fragments by line immunoassay were also negative by ELISA.

### Magnitude of Ab Reactivity Against Ro52 Fragments in SSc and Controls

**Figure 3:**
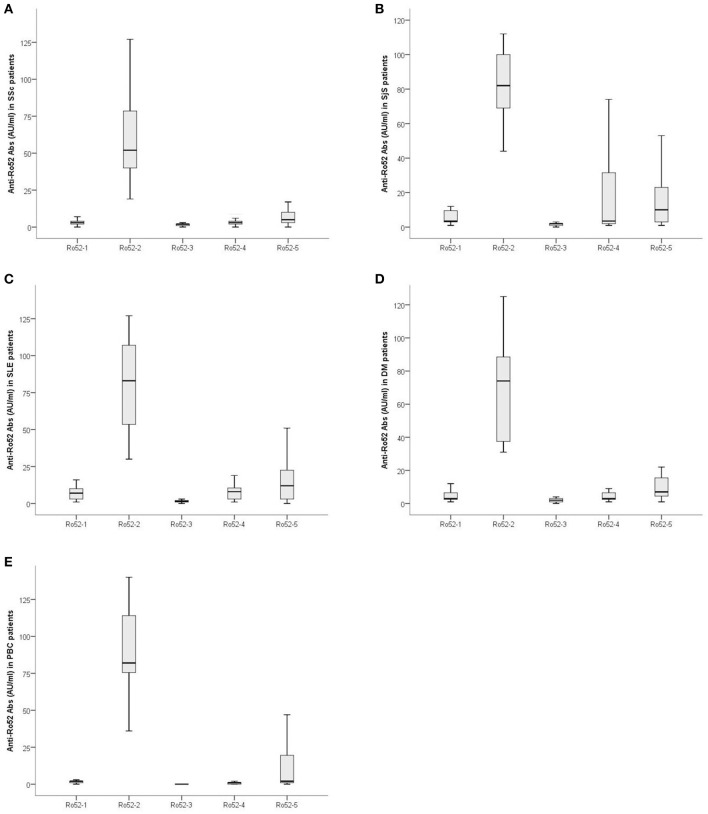
Magnitude of Ab reactivity to individual Ro52 fragments in anti-Ro52-Ab-positive patients: **(A)** 43 patients with Systemic Sclerosis (SSc), **(B)** 20 patients with Sjögren's syndrome (SjS), **(C)** 28 patients with systemic lupus erythematosus (SLE), **(D)** 15 patients with dermatomyositis (DM) and **(E)** 15 patients with primary biliary cholangitis (PBC). Values are given as box plots which represent interquartile ranges and the solid black line at the approximate center of each box is the median. The arms of each box extend with their ends corresponding to 10 and 90% of the value.

The magnitude of Ab reactivity to individual Ro52 fragments is illustrated in Figure 3. Ab reactivity to Ro52-2 was lower in SSc compared to SjS (59.62 ± 26.21 AU/ml vs. 78.75 ± 25.66 AU/ml; *p* = 0.009), SLE (79.46 ± 30.92 AU/ml; *p* = 0.007), and PBC (90.06 ± 32.31 AU/ml, *p* = 0.004) patients. Reactivity against Ro52-4 was lower in SSc (4.88 ± 5.75 AU/ml) than in PBC (1.26 ± 2.15 AU/ml; *p* = 0.001) (Figure 3).

#### Ro52 Epitope Recognition in anti-Ro52+/anti-Ro60+ (double Positive) and anti-Ro52+/anti-Ro60- Patients

When anti-Ro52 Ab-positive patients were divided in anti-Ro52+/anti-CEN+ and anti-Ro52+/anti-CEN- no differences were found in epitope recognition patterns. Similarly, comparisons between subgrouping anti-Ro52+/anti-Scl70+ and anti-Ro52+/anti-Scl70- did not reveal statistically significant differences.

When anti-Ro52 positive patients were divided in anti-Ro52+/anti-Ro60+ (double positive) and anti-Ro52+/anti-Ro60–, statistically significant differences amongst diseases were found. In SSc patients (*n* = 43), reactivity against Ro52-1 was more frequent in anti-Ro52+/anti-Ro60+ patients than anti-Ro52+/anti-Ro60– (5/15 [33.3%] vs. 0/28 [0%], *p* = 0.003) (Table 4).

**Table 4:** Ab reactivity against Ro52 fragments in patients subdivided toanti-Ro52+/anti-Ro60+ and anti-Ro52+/anti-Ro60–.

**Ro52**	**SSc Ro52/Ro60+ (*n* = 15) (%)**	**SSc Ro52+/Ro60- (*n* = 28) (%)**	**p**	**SjS Ro52/Ro60+ (*n* = 14) (%)**	**SjS Ro52/Ro60- (*n* = 6) (%)**	**p**	**SLE Ro52+/Ro60+ (*n* = 18) (%)**	**SLE Ro52+/Ro60- (*n* = 10) (%)**	**p**	**DM Ro52+/Ro60+ (*n* = 3) (%)**	**DM Ro52+/Ro60- (*n* = 12) (%)**	**P**	**PBC Ro52+/Ro60+ (*n* = 2) (%)**	**PBC Ro52+/Ro60- (*n* = 13)**	**P**
Full-length Ro52	100	100	–	100	100	–	100	100	–	100	100	–	100	100	–
Ro52-1	33.3	0	**0.003**	50.0	16.7	NS	33.3	60.0	NS	33.3	16.7	NS	0	7.8	NS
Ro52-2	100	100	–	100	100	–	100	100	–	100	100	–	100	100	–
Ro52-3	0	0	–	0	0	–	0	0	–	0	0	–	0	0	–
Ro52-4	33.3	25.0	NS	50.0	16.7	NS	55.6	60.0	NS	33.3	33.3	NS	0	7.8	NS
Ro52-5	60.0	57.1	NS	71.4	33.3	NS	55.6	60.0	NS	33.3	41.7	NS	50.0	38.5	NS

*SSc, systemic sclerosis; SjS, Sjögren's syndrome; SLE, systemic lupus erythematosus; PBC, primary biliary cholangitis. Significant p-values are indicated in bold*.

Comparing reactivity against various Ro52 fragments in anti-Ro52+/anti-Ro60+ patients among various diseases, no statistically significant difference was detected. On the contrary, the same comparison in anti-Ro52+/anti-Ro60– patients among various diseases showed that reactivity against Ro52-1 was less frequent in SSc compared to SLE (0/28 [0%] vs. 6/10, [60%], *p* = 0.001); Similarly, reactivity against Ro52-4 was less frequent in SSc than in SLE (5/28 [17.9%] vs. 6/10 [60%], *p* = 0.019) patients.

In anti-Ro52-positive SSc patients, the clinical and immunological characteristics between anti-Ro52+/anti-Ro60– and anti-Ro52+/anti-Ro60+ SSc patients did not reveal any statistically significant differences (Supplementary Table 2). Similarly, clinical and immunological features were not statistically different when SSc patients were divided according to reactivity to specific Ro52 (Ro52-1, Ro52-4, Ro52-5) fragments.

## Discussion

This is the first comprehensive analysis of B-cell epitope mapping of anti-Ro52 Abs in patients with SSc using large polypeptidyl fragments spanning the whole Ro52 antigen. Our data show that, as in other AIRDs, such as SjS and SLE (13–19), the dominant epitopic region universally recognized by anti-Ro52 Abs in SSc is that lying within the coiled coil domain of the protein (aa 125-268) (13, 21). Lack of Ab binding of a sequence spanning the C-terminus of the antigen, reported in SjS and SLE is also confirmed in the present study (13–19, 21). However, our study revealed novel findings: patients with SSc less frequently recognize Ro52-1 compared to SLE patients. More importantly, anti-Ro60+ SSc patients showed a distinct, previously unrecognized epitopic pattern, characterized by broad recognition of Ro52 epitopes (including Ro52-1, Ro52-2, Ro52-4, and Ro52-5) compared to anti-Ro60- SSc patients where reactivity by large is restricted to Ro52-2.

In particular, SSc sera were less frequently reactive to Ro52-1 -the N-terminus fragment spanning aa 1-127—than combined SjS/SLE sera (11.6 vs. 41.7%). In a similar vein, Abs against Ro52-4 (aa 57-180)—which partly overlaps with Ro52-1 were less frequently found in SSc than in SjS/SLE (27.9 vs. 50%). This led us to assume that, while the overlapping region contains an epitope (or epitopes) of anti-Ro52 in SjS and SLE such an epitope recognition is absent, at least in part in SSc. Why Ab responses against specific Ro52 fragments are different in frequency and strength among various autoimmune diseases is not an easy task to address (13, 35–38). We can only speculate that the exact mechanisms which are responsible for the induction of anti-Ro52 Ab responses in SSc somewhat differ from those operating in SLE and SjS. It should be noted that in SSc anti-Ro52 Abs less frequently co-exist with anti-Ro60 Abs compared to SLE and SjS, which usually have both autoAb specificities (8, 10, 39). A similar to SSc pattern of less frequently recognition of Ro52-1 and Ro52-4 was also seen in PBC, suggesting that a common (or similar) mechanism of autoAbs production for both diseases may be in operation (40).

The increased frequency of reactivity against Ro52-1 and Ro52-5 in anti-Ro52/anti-Ro60 double positive patients than in anti-Ro52+/anti-Ro60– SSc patients is difficult to explain. Currently, it is not known why some patients have reactivities to Ro52 alone, Ro60 alone or both (41). The two autoantigens are structurally unrelated but—immunologically—interrelated since anti-Ro52 and anti-Ro60 immune responses tend to co-exist (42, 43). However, anti-Ro52 autoAbs can be present without ever anti-Ro60 reactivity in many autoimmune diseases. Mechanisms, such as epitope spreading and exposure to cryptic epitopes in double positive sera at very early stages of disease may account for con-current reactivity (35–37, 44, 45). The clinical significance of epitopic recognition is underlined in experimental diseases, where the clinical phenotype largely depends on the Ro52 domain, used as an immunogen. For instance, Sroka et al. (46) have recently shown that only immunization with the coiled coil Ro52 domain and its subsequent immune response against the coiled coil Ro52 domain can induce salivary gland dysfunction (46). However, we were unable to find specific associations between clinical features and epitope profiling. The relatively recent demonstration of the true nature of the Ro52 antigen and its pleiotropic key role for signal transduction, in adaptive and innate immunity as member of the TRIM family of proteins may explain (at least in part) some of these attributes (47–49). Ro52 (TRIM21) is an intra-cytoplasmic receptor of IgG and epitope spreading mechanisms involving regions corresponding to dominant or subdominant epitopes of Ro52 and Ro60 may account for the observed distinct Ab recognition against the two antigens (11, 12).

Our data suggests that epitope mapping of anti-Ro52 Abs in systemic sclerosis reveals a common denominator, the coiled-coil related epitope which, similarly to SjS, SLE, and other autoimmune diseases, is universally reactive. The N-terminus region spanned by aa 57-180 is a dominant epitope in anti-Ro52+/Ro60+ SSc patients but not in Ro52+/Ro60- SSc patients suggesting that Ro60 directly or indirectly is involved in the shaping of the epitopic repertoire of anti-Ro52 Abs, a finding which warranties further investigation(35, 36). Understanding the mechanisms responsible for the breaking of tolerance to Ro52 in SSc may shed a light not only for the understanding of the pathogenesis of this disease but also for those of SjS and SLE, positioning Ro60 as a key player. By no means our study or other studies of this kind (21) can address the key question that arises. Are these data epiphenomenal or do they really play a role in the induction of anti-Ro52 or anti-Ro60 in SSc, other AIRDs or indeed in other autoimmune diseases? Nevertheless, our data provide the impetus for subsequent studies performed in serum samples from patients on a large scale, as well as in experimental models of the disease.

## Author Contributions

Each authors named as an author has made substantial contributions to the conception, design of the study, or acquisition, analysis, and interpretation of data. AG, CL, MGM performed experiments; TSc, WM prepared antigenic preparations; TS, CK, DD and LIS performed clinical assessments; TS, AG, AT, and CK prepared clinical and laboratory datasets; AG and CL analyzed the data; AG, LIS and DPB drafted the manuscript; DPB and LIS supervised the project and designed the experimental work; DPB had the original idea. All authors approved the final version of the manuscript.

### Conflict of Interest Statement

TSc and WM are employees of Euroimmun, Germany. The remaining authors declare that the research was conducted in the absence of any commercial or financial relationships that could be construed as a potential conflict of interest.

## Supplementary Material

Supplementary Figure 1 Correlation of line immunobloding readings and ELISA absorbance values testing of individual fragments from patients with systemic sclerosis. The immunobloting data were confirmed in a representative set of sera from 12 anti-Ro52 Ab positive SSc patients and 20 anti-Ro52 Ab negative controls (Data not included). A statistically significant correlation is noted for all fragments between the two assays readouts.

Supplementary Table 1 Representation of the alternative cut off calculation, by the mean plus two standard deviations of the reactivity values of anti-Ro52 negative patients with autoimmune rheumatic diseases.

Supplementary Table 2 Main demographic, clinical and immunological parameters in anti- Ro52+/Ro60+ and Ro52+/Ro60- patients with SSc.

## Abbreviations

Ab: antibody
AutoAb: autoantibody
ARD: autoimmune rheumatic diseases
PBC: primary biliary cholangitis; Sjögren's syndrome
SLE: systemic lupus erythematosus
SSc: systemic sclerosis.
