# Supplementary figures and images for: Anti-Ro60 Seropositivity Determines Anti-Ro52 Epitope Mapping in Patients With Systemic Sclerosis
Source: Front Immunol. 2018 Dec 7;9:2835. doi: 10.3389/fimmu.2018.02835 (PMC6293197; doi:10.3389/fimmu.2018.02835)

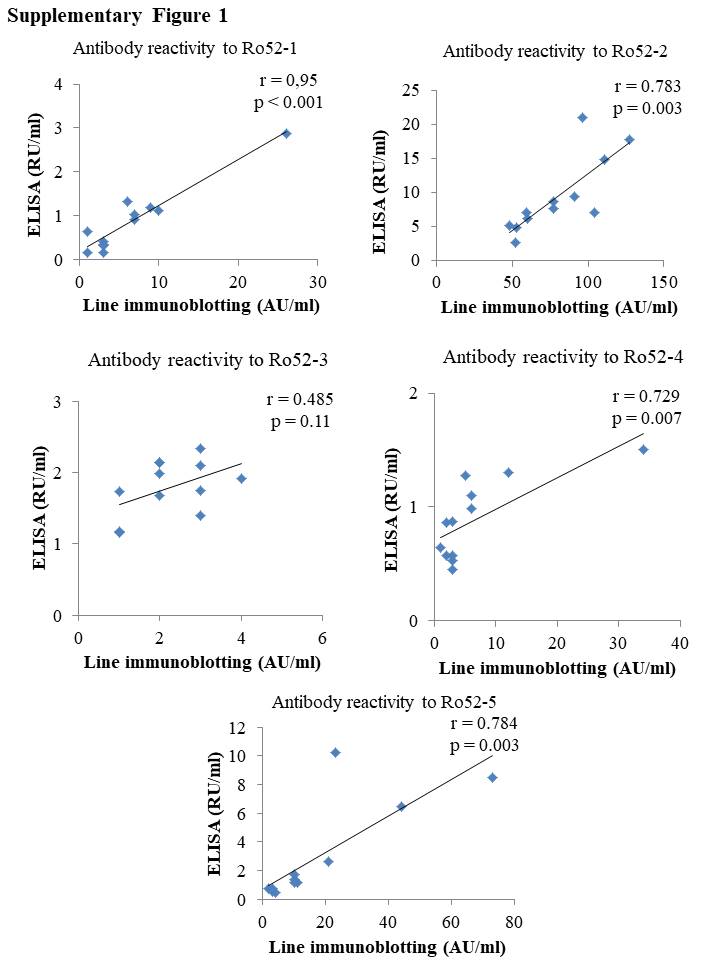

Supplement: Supplementary Figure 1 — Correlation of line immunobloding readings and ELISA absorbance values testing of individual fragments from patients with systemic sclerosis. The immunobloting data were confirmed in a representative set of sera from 12 anti-Ro52 Ab positive SSc patients and 20 anti-Ro52 Ab negative controls (Data not included). A statistically significant correlation is noted for all fragments between the two assays readouts. [file Image_1.JPEG]
