# Supplementary material for: Anti-Ro60 Seropositivity Determines Anti-Ro52 Epitope Mapping in Patients With Systemic Sclerosis
Source: Front Immunol. 2018 Dec 7;9:2835. doi: 10.3389/fimmu.2018.02835 (PMC6293197; doi:10.3389/fimmu.2018.02835)
Supplement: Supplementary Table 1 — Representation of the alternative cut off calculation, by the mean plus two standard deviations of the reactivity values of anti-Ro52 negative patients with autoimmune rheumatic diseases. [file Table_1.DOCX]

**Supplementary Table 1**. Representation of the alternative cut off calculation, by the mean plus two standard deviations of the reactivity values of anti-Ro52 negative patients with autoimmune rheumatic diseases.

|  | Ro52-1 | Ro52-2 | Ro52-3 | Ro52-4 | Ro52-5 |
| --- | --- | --- | --- | --- | --- |
| mean + 2sd (AU/ml) | 3.05 + 2x2.23 = 7.51 | 3.45 + 2x3.03 = 9.51 | 1.3 + 2x1.01 = 3.32 | 2.1 + 2x1.3 = 4.7 | 2.4 + 2x2.37 = 7.14 |
| Cut off (AU/ml) | 8 | 10 | 4 | 5 | 8 |

|  | **Ro52+/Ro60- n=19** | **Ro52+/Ro60+ n=13** | **p** |
| --- | --- | --- | --- |
| **Sex** |  |  |  |
| **Female** | 17 (89,5) | 13 (100) | ns  ns |
| **Male** | 2 (10,5) | 0 (0) |  |
| **Age (mean±SD)** | 54.03 ± 13.53 | 57.43 ± 9.80 | ns |
| **SSc type** |  |  |  |
| **lcSSc (n,%)** | 11 (57.9) | 9 (69.2) | ns |
| **dcSSc (n,%)** | 8 (42.1) | 4 (30.8) | ns |
| **Pulmonary fibrosis (n,%)** | 7 (36.8) | 4 (30.8) | ns |
| **Pulmonary Arterial Hypertension (n,%)** | 2 (10.53) | 2 (15.4) | ns |
| **Ulcers (n,%)** | 5 (26.3) | 7 (53.8) | ns |
| **Disease Duration (mean±SD)** | 11.05 ± 9.6 | 14.23 ± 8.7 | ns |
| **RSS (mean±SD)** | 9.52 ± 9.08 | 5.58 ± 7.29 | ns |
| **GI involvement** |  |  |  |
| **Upper (n,%)** | 10 (52.6) | 8 (61.5) | ns |
| **Lower (n,%)** | 0 | 0 | na |
| **Both (n,%)** | 1 (5.3) | 0 | ns |
| **Arthritis (n,%)** | 5 (26.3) | 7 (53.8) | ns |
| **Serositis (n,%)** | 0 | 3 (23.1) | ns |
| **Telangiectasia (n,%)** | 11 (57.9) | 5 (38.5) | ns |
| **CPK (n,%)** | 1 (5.3) | 0 | ns |
| **Calcinosis (n,%)** | 2 (10.53) | 0 | ns |
| **Renal Crisis (n,%)** | 0 | 0 | ns |
| **Overlap Syndrome/MCTD (n,%)** | 5 (26.3) | 3 (23.1) | ns |
| **Dry mouth (n,%)** | 6 (31.6) | 6 (46.2) | ns |
| **Dry eyes (n,%)** | 4 (21.05) | 3 (23.1) | ns |
| **Rash (n,%)** | 4 (21.05) | 3 (23.1) | ns |
| **Acroosteolysis (n,%)** | 4 (21.05) | 1 (7.7) | ns |
|  |  |  |  |
| **Autoantibodies** | |  |  |
| **-Scl-70 (n,%)** | 3 (15.8) | 2 (15.4) | ns |
| **-CENPA (n,%)** | 8 (42.1) | 4 (30.8) | ns |
| **-CENPB (n,%)** | 8 (42.1) | 4 (30.8) | ns |
| **-RP11 (n,%)** | 1 (5.3) | 0 | *ns* |
| **-RP155 (n,%)** | 3 (15.8) | 0 | ns |
| **-Fibrillarin (n,%)** | 0 | 1 (7.7) | ns |
| **-NOR90 (n,%)** | 2 (10.5) | 1 (7.7) | ns |
| **-Th/To (n,%)** | 0 | 0 | na |
| **-PM-Scl 100 (n,%)** | 0 | 0 | na |
| **-PM-Scl 75 (n,%)** | 1 (5.3) | 1 (7.7) | ns |
| **-Ku (n,%)** | 4 (21.1) | 0 | ns |
| **-PDGFR (n,%)** | 0 | 0 | na |
| **-Ro52 (n,%)** | 19 (100) | 13 (100) | na |
| **-Ro60 (n,%)** | 0 | 13 (100) | na |
| **-La (n,%)** | 0 | 5 (38.5)) | 0.006 |

**Supplementary Table** **2.** Main demographic, clinical and immunological parameters in anti- Ro52+/Ro60+ and Ro52+/Ro60- patients with SSc
